# The association between large language model dependence and academic anxiety among nursing students: statistical indirect associations through artificial intelligence addiction and academic burnout

**DOI:** 10.3389/fpsyg.2026.1917190

**Published:** 2026-10-27

**Authors:** Taiping Li, Qiuxian Ye, Jifeng Dong, Yanqing Yan

**Affiliations:** 1 Guangdong University of Science and Technology, Dongguan, Guangdong, China; 2 Zhuhai College of Science and Technology, Zhuhai, Guangdong, China

**Keywords:** academic anxiety, academic burnout, artificial intelligence addiction, large language model dependence, nursing students

## Abstract

**Background:**

Increasing use of large language models in nursing education has raised questions about the association between reliance on these tools and academic distress. This study examined direct and specific indirect associations between large language model dependence (LMD) and academic anxiety (AA) through artificial intelligence addiction (AIA) and academic burnout (AB).

**Methods:**

A cross-sectional survey was conducted from March to April 2026 among 473 nursing students in Guangdong Province, China, including students undertaking clinical placements. The four constructs were assessed using items selected from published self-report instruments. Partial least squares structural equation modeling was performed with age and sex included as covariates. Direct and specific indirect associations were evaluated using bootstrapping with 5,000 resamples.

**Results:**

Cronbach’s α ranged from 0.914 to 0.944, composite reliability from 0.935 to 0.954, and average variance extracted from 0.560 to 0.721. Heterotrait–monotrait ratios ranged from 0.485 to 0.733, supporting discriminant validity. Model fit evidence was mixed: SRMR was 0.068, whereas NFI was 0.812, below the 0.90 reference value. The model explained 49.2% of the variance in academic anxiety. Large language model dependence showed a small positive direct association with academic anxiety (*β* = 0.138, 95% CI [0.006, 0.278], *p* = 0.048). Significant specific indirect associations were identified through academic burnout (*β* = 0.205, 95% CI [0.123, 0.303]) and through artificial intelligence addiction followed by academic burnout (*β* = 0.098, 95% CI [0.049, 0.147]); both *p* < 0.001.

**Conclusion:**

Large language model dependence was positively associated with academic anxiety among nursing students, with specific indirect associations through academic burnout and through artificial intelligence addiction followed by academic burnout. These findings highlight the relevance of problematic AI use and study-related exhaustion to academic distress. However, the cross-sectional design does not establish temporal ordering or causal mediation.

## 1 Introduction

The rapid development of artificial intelligence has reshaped the learning, information seeking and professional practice in educational and healthcare settings. Among the emerging AI technologies, large language models have attracted much attention because of their ability to generate text, interpret information, summarize complex materials, support writing, and quickly respond to user queries (Gunawan et al., 2024). In nursing education and practice, such tools can provide practical support for academic learning, clinical preparation, knowledge retrieval and task completion (Hobensack et al., 2024). For nursing students who often face heavy academic demands, professional pressure (Andargeery et al., 2025; Mohamed et al., 2024), and complex knowledge needs, large language models may serve as convenient and efficient resources. However, the increasing use of these tools also raises concerns about whether repeated reliance on AI-generated responses may be associated with psychological dependence or problematic patterns of use (Sallam, 2023).

Large language model dependence refers to a tendency to rely on large language models to complete cognitive, academic, or work-related tasks, especially when users believe that these tools can satisfy most of their expected functions (Yankouskaya et al., 2025). Although appropriate use of large language models may improve efficiency, excessive dependence may be associated with lower willingness to engage in independent thinking, problem-solving, and self-regulated learning (Bastani et al., 2025; Tian and Zhang, 2025). This issue is particularly important in nursing-related populations, because nursing education and clinical practice not only need to acquire knowledge, but also need critical thinking, professional judgment, emotion regulation and autonomous decision-making (Sharifi Kelarijani et al., 2024). When individuals rely excessively on AI tools, they may encounter difficulties in managing academic tasks without technical help, which may be linked to greater psychological vulnerability in an educational or professional environment.

Another concern is that dependence on large language models may be related to AI addiction. AI addiction reflects a maladaptive pattern of AI use, which is characterized by long use of AI tools, difficulty in controlling use, and continued participation despite possible negative consequences (El-Sayed et al., 2025). As large language models become more accessible and responsive, users may increasingly turn to them for academic, emotional, or practical support. This reuse may be related to stronger habitual dependence and greater possibility of problematic AI engagement (Maral et al., 2025). For nursing students, overuse of AI may be related to low learning enthusiasm and additional psychological pressure when AI tools are unavailable or insufficient.

Academic burnout is another important psychological concern among nursing-related populations (Olson et al., 2025). Nursing students usually experience high levels of academic and professional demands, including intensive courses, clinical training, emotional labor and performance expectations (Andargeery et al., 2025). Academic burnout is usually manifested as emotional exhaustion, reduced motivation and the feeling of being overwhelmed by academic or learning-related responsibilities (Kong et al., 2023; Qin et al., 2025). When individuals use AI tools as coping strategies, but fail to develop deeper understanding, effective learning habits or sustainable self-management skills, problematic AI use may be associated with burnout. In this sense, AI addiction may not only represent the frequent use of AI, but also indicate a maladaptive pattern related to learning fatigue and the reduction of psychological resources.

Academic anxiety is also an important issue in nursing education and practice. Academic anxiety includes anxiety, tension and negative emotional reactions related to academic achievement, academic adequacy and educational needs (Mohamed et al., 2024). In a highly demanding nursing environment, academic anxiety may be higher when individuals are uncertain about their ability, learning outcomes, or ability to meet professional expectations (García-Velasco et al., 2025; Sarfika et al., 2025; Seshabela and Shakwane, 2024). Dependence on large language models may be directly related to academic anxiety, because individuals who rely heavily on AI tools may lack confidence in their learning ability (Liu et al., 2026). At the same time, this relationship may also operate indirectly through AI addiction and academic burnout (Elshaer and Azazz, 2025). Specifically, strong dependence on large language models may be linked to greater problematic AI use, which may in turn be associated with higher academic burnout and academic anxiety.

Although research on AI-related reliance and problematic engagement is expanding, their relationships with academic distress warrant further examination in nursing education. A central question is whether large language model dependence and artificial intelligence addiction provide distinct information about students’ academic experiences (El-Sayed et al., 2025; Li et al., 2025). Another is whether academic burnout is involved in specific statistical indirect associations with academic anxiety. Examining these constructs within a single model may help distinguish the associations involving problematic AI engagement from those involving study-related exhaustion, while recognizing that their temporal ordering remains unresolved.

This study examined direct and specific statistical indirect associations among large language model dependence, artificial intelligence addiction, academic burnout, and academic anxiety in nursing students, including those undertaking clinical placements. Using partial least squares structural equation modeling, we evaluated the indirect association between large language model dependence and academic burnout through artificial intelligence addiction, and the indirect associations with academic anxiety through academic burnout and through artificial intelligence addiction followed by academic burnout. The proposed ordering was theoretically specified; the cross-sectional design did not permit conclusions about temporal precedence or causal mediation. The study aimed to clarify how these distinct constructs were associated within the nursing education context.

## 2 Literature review and research hypotheses

### 2.1 Theoretical foundation

This study draws on perspectives on problematic technology use and social stress. Research on large language model dependence conceptualizes reliance on AI assistance as a measurable aspect of human–AI interaction (Li et al., 2025). Research on artificial intelligence addiction distinguishes problematic engagement through features such as compulsive use, overdependency, and functional impairment (El-Sayed et al., 2025). These perspectives provide a basis for examining large language model dependence and artificial intelligence addiction as related but distinct constructs. Social stress theory further emphasizes the relevance of stressors and coping resources to psychological distress (Aneshensel, 1992), informing the examination of academic burnout and academic anxiety in nursing education.

Together, these perspectives guide a model linking AI-related reliance, problematic AI engagement, and academic distress among nursing students, including those undertaking clinical placements. The model examines whether the associations are consistent with the proposed theoretical ordering. It does not assume that reliance necessarily develops into addiction or that addiction, burnout, and anxiety occur in a fixed temporal sequence.

### 2.2 Large language model dependence and artificial intelligence addiction

Large language models have been increasingly embedded in academic learning, information acquisition and professional support. For nursing students, these tools can provide immediate help in understanding complex knowledge, preparing for academic tasks and coping with learning related difficulties. However, when users repeatedly rely on large language models to reduce cognitive efforts or solve academic problems, this use may be linked to a shift from functional assistance to maladaptive dependence. The greater dependence on language models reflects a strong tendency to rely on responses generated by AI to complete learning or work-related tasks, which may be associated with users’ perceived needs for independent judgment and self-directed problem solving (Montag et al., 2025).

From the perspective of problematic technology use, dependence on digital tools may be associated with a greater likelihood of uncontrolled and excessive engagement. Because large language models provide fast, personalized, and easily accessible responses, users with strong dependencies may prefer to use these tools beyond their original purpose or expected duration. This repeated pattern may be related to stronger habitual use, lower self-regulation and a greater likelihood of AI addiction. This problem may be particularly prominent among nursing-related populations, because academic and clinical needs may encourage individuals to rely on AI tools as a convenient coping strategy (Al-Obaydi and Pikhart, 2026; Sağlam and Kalanlar, 2025). Therefore, this study proposes the following assumptions:

*H1*: Large language model dependence is positively associated with artificial intelligence addiction.

### 2.3 Artificial intelligence addiction and academic burnout

AI addiction refers to a problematic mode of AI use, which is characterized by excessive participation, difficulty in controlling use, and continued reliance on AI despite possible negative consequences. Although AI tools can support learning and work efficiency, addictive or poorly managed AI use may be associated with lower effective academic participation. When individuals spend too much time using AI tools or rely heavily on AI tools to complete academic tasks, excessive or poorly managed use of AI may be associated with less active learning, less reflective thinking and weaker deep knowledge processing ability. This maladaptive pattern may be related to higher psychological pressure and lower academic motivation (Farghaly Abdelaliem et al., 2022).

Academic burnout usually manifests as emotional exhaustion, reduced enthusiasm and the feeling of being overwhelmed by academic requirements. In nursing education and practice, individuals often face intensive courses, clinical training, professional expectations and emotional pressure. If AI is overused, it may not be able to effectively reduce these requirements; On the contrary, it may be associated with additional dependencies, uncertainties and pressures. When users need to complete tasks without AI support, they may lack confidence in their learning ability and feel more tired (Ali et al., 2025; Sağlam and Kalanlar, 2025). Therefore, AI addiction may be related to higher levels of academic burnout. Accordingly, the following assumptions are proposed in this study:

*H2*: Artificial intelligence addiction is positively associated with academic burnout.

### 2.4 Academic burnout and academic anxiety

Academic burnout is a state of psychological exhaustion caused by long-term academic or learning related needs. Individuals experiencing academic burnout may feel emotional exhaustion, lack of motivation, and lack of ability to deal with academic responsibility. Academic burnout may be particularly important among nursing-related groups, because nursing students need theoretical knowledge and practical ability of management. Continuous exposure to academic pressure, clinical expectations and performance requirements may be associated with reduced psychological resources and greater emotional vulnerability (Hwang and Kim, 2022).

Academic anxiety refers to anxiety, tension and negative emotional reactions related to academic achievement, perceived inadequacy and uncertainty of learning outcomes. When individuals experience burnout, they may think that academic tasks are more threatening and difficult to manage. Emotional exhaustion and reduced academic engagement may be associated with weaker confidence in coping with academic challenges and stronger anxiety (Fard et al., 2020; Ghattas and El-Ashry, 2024; Zhu et al., 2023). In this sense, academic burnout may be an important psychological correlate of academic anxiety. Therefore, this study proposes the following assumptions:

*H3*: Academic burnout is positively associated with academic anxiety.

### 2.5 Large language model dependence and academic anxiety

Large language model dependence may also be directly related to academic anxiety. Although large language models can provide academic support, excessive dependence may be associated with weaker confidence in individuals’ knowledge, judgment, and ability to solve problems independently. When users rely heavily on the responses generated by AI, they may not be sure whether they can complete academic tasks without technical help. This uncertainty may be particularly relevant in nursing education and practice, because professional ability, critical thinking and accurate judgment are essential (Ma et al., 2025; Ye et al., 2025).

In addition, when users encounter complex academic tasks, unreliable AI output or unavailability of AI support, their dependence on large language models may be associated with greater psychological vulnerability. In this case, individuals with strong dependence may have greater concerns about academic performance and personal adequacy (Elshaer and Azazz, 2025). Therefore, large language model dependence may not only be linked to problematic AI use, but may also be directly associated with academic anxiety. Accordingly, this study proposes the following hypothesis:

*H4*: Large language model dependence is positively associated with academic anxiety.

### 2.6 Hypothesized statistical indirect associations

Artificial intelligence addiction may statistically link large language model dependence with academic burnout. Large language model dependence concerns reliance on AI assistance, whereas artificial intelligence addiction encompasses problematic engagement, including compulsive use and functional impairment (El-Sayed et al., 2025; Li et al., 2025). This distinction motivates the examination of whether reliance is indirectly associated with study-related exhaustion through difficulties regulating AI engagement. This proposed indirect association is a theoretical extension of the construct definitions, rather than an established consequence of dependence.

Academic burnout may also statistically link large language model dependence with academic anxiety. From a social stress perspective, psychological distress should be considered in relation to stressors and coping resources (Chang and Hou, 2022). In the present model, burnout represents study-related exhaustion, whereas anxiety concerns academic demands and performance (Hwang and Kim, 2022). Their conceptual relationship provides a rationale for examining burnout as an intermediate construct in the association between dependence and academic anxiety.

The model additionally specifies an indirect association through artificial intelligence addiction followed by academic burnout. Addiction is positioned first because it concerns the regulation of AI engagement, while burnout concerns exhaustion in the academic context (Hwang and Kim, 2022). This ordering represents the hypothesis that problematic engagement may be associated with study-related exhaustion, which is associated with academic anxiety. It is theoretically proposed and remains open to alternative orderings. Accordingly, the following hypotheses concern statistical indirect associations:

*H5*: Large language model dependence is positively indirectly associated with academic burnout through artificial intelligence addiction.

*H6*: Large language model dependence is positively indirectly associated with academic anxiety through academic burnout.

*H7*: Large language model dependence is positively indirectly associated with academic anxiety through artificial intelligence addiction followed by academic burnout.

Figure 1 presents the hypothesized direct and statistical indirect associations among the study constructs.

**Figure 1:**
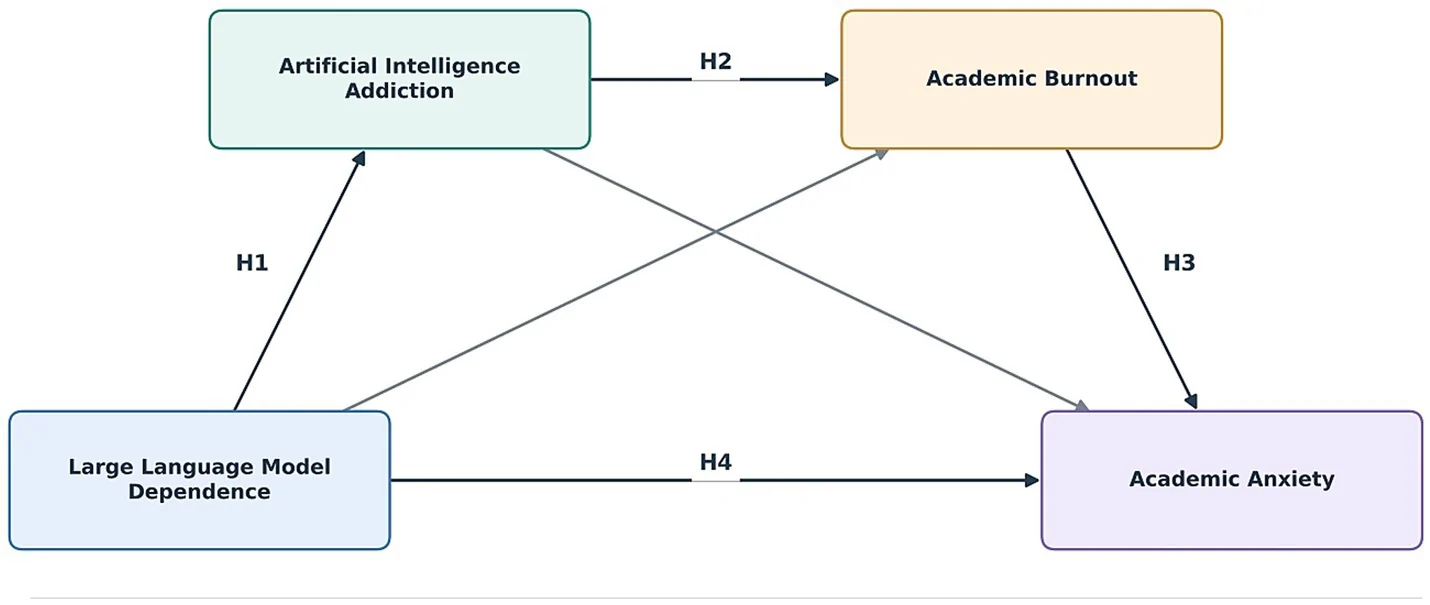
Hypothesized model of direct and indirect associations among the study constructs. H1–H4 denote the hypothesized direct associations. The hypothesized indirect associations are H5: LMD → AIA → AB; H6: LMD → AB → AA; and H7: LMD → AIA → AB → AA. Unnumbered arrows represent additional direct paths included in the model. LMD, large language model dependence; AIA, artificial intelligence addiction; AB, academic burnout; AA, academic anxiety.

## 3 Methods

### 3.1 Participants

All participants were enrolled nursing students, including those undertaking clinical placements as part of their nursing education. Students undertaking clinical placements retained their student status. The sample therefore represented nursing students at different stages of their education, encompassing both classroom learning and clinical placement experiences.

A cross-sectional survey was conducted from March to April 2026 using convenience sampling. Nursing students were recruited from higher education institutions in Guangdong Province, China, including students undertaking clinical placements in hospitals in the province. All participants retained their student status. The research team contacted teaching staff, clinical placement supervisors, and relevant coordinators to facilitate recruitment. Questionnaire links were distributed through student groups, placement-related communication groups, and relevant online channels. Participants received information about the study purpose, voluntary participation, and confidentiality before completing the questionnaire.

A total of 500 nursing students were invited to participate. After screening and excluding invalid questionnaires, including incorrectly completed questionnaires and duplicate submissions, 473 valid questionnaires were retained for the final analysis, representing 94.6% of those invited. Participation was voluntary, and informed consent was obtained before questionnaire completion.

The inclusion criteria were: (a) current enrollment in a nursing education program, including students undertaking clinical placements; and (b) voluntary agreement to participate after reading the study information and providing informed consent.

A total of 473 valid respondents were included in the analysis. Among them, 19.7% were male and 80.3% were female. In terms of age distribution, 43.55% were aged 18–20 years, 46.09% were aged 21–23 years, 6.34% were aged 24–26 years, 2.33% were aged 27–29 years, and 1.69% were aged 30 years and above.

### 3.2 Measures

All instruments were administered in Chinese. For the scale originally developed in English, a forward-backward translation process was performed. First, two bilingual researchers independently translated English words into Chinese. The preliminary Chinese version was then back-translated into English by another bilingual translator who was unaware of the original text. The research group discussed and resolved the differences to ensure semantic equivalence and cultural appropriateness. A small-scale pilot test was conducted among nursing-related participants to confirm clarity and comprehensibility. In the case of relevant Chinese adaptation materials, these materials are referred to in the process of translation and adaptation.

All instruments used in this study were previously published self-report measures with reported psychometric evidence. No new questionnaire was developed for the present study. The questionnaire consisted of two parts: demographic information and research-related measures. The following section reports the names, sources, and representative items of the instruments. Each research-related measure was assessed using a five-point Likert scale ranging from 1 (strongly disagree) to 5 (strongly agree).

#### 3.2.1 Large language model dependence

Large language model dependence was assessed using 11 items selected from the instrument developed by Li et al. (2025). Items were selected for their relevance to the nursing education context, while preserving their original meaning. An example item was “I believe that large language models possess all the functions I expect.” Cronbach’s α was 0.923 in the present sample.

#### 3.2.2 Artificial intelligence addiction

Artificial intelligence addiction was assessed using nine items selected from the instrument developed by El-Sayed et al. (2025). Although the original instrument was developed for researchers, the selected items were considered relevant to AI use among nursing students. Item selection was guided by the study context, without changing the original meaning of the items. An example item was “I often use AI tools for longer or more frequently than I originally intended.” Cronbach’s α was 0.928 in the present sample.

#### 3.2.3 Academic anxiety

Academic anxiety was assessed using six items selected from the Academic Anxiety Scale developed by Bülbül and Odacı (2025). Items were selected for their relevance to the academic experiences of nursing students, while preserving their original meaning. An example item concerned feelings of guilt about academic inadequacy. Cronbach’s α was 0.914 in the present sample.

#### 3.2.4 Academic burnout

Academic burnout was assessed using eight items selected from the Academic Burnout Scale developed by Jan and Parveen (2024). Items were selected for their relevance to study-related burnout among nursing students, including those undertaking clinical placements, without changing their original meaning. An example item was “My studies leave me feeling drained and emotionally exhausted.” Cronbach’s α was 0.944 in the present sample.

### 3.3 Data analysis strategy

SPSS 29.0 was used to conduct descriptive statistical analyses. Descriptive statistics were calculated using each participant’s mean item score for each construct. Means and sample standard deviations were reported for large language model dependence, artificial intelligence addiction, academic burnout, and academic anxiety. SmartPLS 4.1 was used to assess the measurement model and structural model. PLS-SEM was selected because the primary aim of this study was predictive and explanatory rather than strictly confirmatory (Sarstedt et al., 2021). In addition, the proposed model includes multiple mediating pathways among psychosocial and behavioral constructs, and the data were collected using self-report Likert-type scales that may not fully satisfy multivariate normality assumptions. Therefore, PLS-SEM was considered appropriate for estimating the structural relationships and mediating effects in the present study.

Following the two-step approach recommended in the PLS-SEM literature, the analysis first assessed the measurement model by examining internal consistency reliability, convergent validity, and discriminant validity. Cronbach’s alpha and composite reliability were used to evaluate the internal consistency reliability of the scores, whereas average variance extracted was used to assess convergent validity evidence. Discriminant validity evidence was examined using the HTMT ratio and the Fornell–Larcker criterion. The structural model was evaluated using bootstrapping with 5,000 resamples. Standardized path coefficients, t values, *p* values, and 95% bootstrap confidence intervals were reported for the direct and specific indirect associations. Statistical significance was evaluated using a two-sided significance level of 0.05, with confidence intervals excluding zero providing evidence of a statistically significant association (Hair et al., 2021). Explanatory power was assessed using the coefficients of determination (*R*^2^) of the endogenous constructs. The contribution of each predictor to explained variance was evaluated using *f*^2^, with values of 0.02, 0.15, and 0.35 serving as conventional reference points for small, medium, and large effects, respectively (Cohen, 2013). Model fit was assessed using the standardized root mean square residual (SRMR) and the normed fit index (NFI), with SRMR below 0.08 and NFI above 0.90 used as reference values. These indices were interpreted alongside the measurement and structural model results, rather than as definitive evidence of model adequacy. Age and sex were included as covariates with direct paths to academic anxiety only.

The structural model examined the associations among large language model dependence, artificial intelligence addiction, academic burnout, and academic anxiety, with age and sex included as covariates. The reported direct and specific indirect associations were therefore adjusted for these demographic characteristics. All participants were enrolled nursing students, including those undertaking clinical placements; thus, student versus employed staff status was not an applicable grouping variable. AI-use frequency, duration, and purpose were not measured and could not be included as covariates.

## 4 Results

### 4.1 Common method bias test

All variables in this study were measured through self-report questionnaires from the same respondents. To mitigate the potential influence of common method bias, several procedural control measures were adopted during the data collection process. First, at the beginning of the questionnaire, the respondents were clearly informed that the study was only for academic purposes, all answers would be kept strictly confidential, and there were no “correct” or “wrong” answers, so as to reduce the social expectation effect. At the same time, respondents were encouraged to answer based on their true feelings, so as to improve the authenticity and reliability of the data. In addition, the presentation order of the questionnaire items was randomized to reduce the systematic bias that may be caused by the order of the answers (Elsamen et al., 2025).

To assess the potential influence of common method bias, Harman’s single-factor test was conducted using all measurement items. The first unrotated factor explained 41.884% of the total variance, which was below the commonly used 50% threshold (Podsakoff et al., 2003), and five factors had eigenvalues greater than 1, suggesting that the variance was not dominated by a single factor. However, because all variables were collected from the same respondents using self-report questionnaires at a single time point, and because the first factor was close to or slightly above some more conservative criteria, common method variance could not be completely ruled out. Therefore, the results should be interpreted with caution.

### 4.2 Measurement model

Table 1 presents the descriptive statistics of the main study variables. The mean scores were 3.336 (SD = 0.675) for large language model dependence, 3.384 (SD = 0.784) for artificial intelligence addiction, 3.017 (SD = 0.881) for academic burnout, and 3.159 (SD = 0.815) for academic anxiety. All four constructs had complete item responses from the 473 participants. As shown in Table 1, the measurement model demonstrated acceptable internal consistency reliability of the scores and convergent validity evidence. Specifically, Cronbach’s *α* values ranged from 0.914 to 0.944, while composite reliability (CR) values ranged from 0.935 to 0.954, with both exceeding the recommended threshold of 0.70. In addition, the average variance extracted (AVE) values ranged from 0.560 to 0.721, all of which were above the minimum cutoff value of 0.50, indicating adequate convergent validity. Most indicators had outer loadings above 0.70. Two indicators, LMD8 and AA1, had lower loadings of 0.672 and 0.601, respectively. Indicators with loadings below 0.70 should be evaluated alongside construct reliability, convergent validity, and content coverage rather than automatically removed (Hair et al., 2021). In the present sample, LMD demonstrated composite reliability of 0.935 and AVE of 0.560, while AA demonstrated composite reliability of 0.936 and AVE of 0.712. Both constructs therefore met the specified construct-level reliability and convergent validity criteria. LMD8 and AA1 were retained in the reported model, although their lower loadings indicate weaker indicator reliability and warrant caution when interpreting measurement quality. The decision to retain these indicators also considered their relevance to the intended construct content and the potential narrowing of the selected item sets through further deletion. Consequently, the measurement model provided acceptable convergent validity evidence (Hair et al., 2019). As reported in Table 2, the HTMT values ranged from 0.485 to 0.733, all of which were below the conservative threshold of 0.85. This provided evidence that the constructs were empirically distinct from one another according to the HTMT criterion (Hair et al., 2021). Furthermore, as shown in Table 3, the square roots of the AVE values (diagonal values) were greater than the correlations between constructs (off-diagonal values). These findings provide additional support for the discriminant validity of the measurement model (Hair et al., 2021).

**Table 1:** Internal consistency reliability and convergent validity evidence.

Constructs	Items	Mean	SD	Outer loadings	Cronbach’*α*	CR	AVE
Large Language Model Dependence	LMD 1	3.336	0.675	0.750	0.923	0.935	0.560
	LMD 2			0.785			
	LMD 3			0.780			
	LMD 4			0.707			
	LMD 5			0.730			
	LMD 6			0.705			
	LMD 7			0.736			
	LMD 8			0.672			
	LMD 9			0.820			
	LMD10			0.750			
	LMD11			0.785			
Artificial Intelligence Addiction	AIA 1	3.384	0.784	0.798	0.928	0.940	0.634
	AIA 2			0.791			
	AIA 3			0.808			
	AIA 4			0.822			
	AIA 5			0.797			
	AIA 6			0.782			
	AIA 7			0.833			
	AIA 8			0.763			
	AIA 9			0.767			
Academic Anxiety	AA 1	3.159	0.815	0.601	0.914	0.936	0.712
	AA 2			0.854			
	AA 3			0.896			
	AA 4			0.898			
	AA 5			0.889			
	AA 6			0.904			
Academic Burnout	AB1	3.017	0.881	0.834	0.944	0.954	0.721
	AB2			0.861			
	AB3			0.892			
	AB4			0.849			
	AB5			0.862			
	AB6			0.758			
	AB7			0.862			
	AB8			0.869			

**Table 2:** Discriminant validity evidence based on the HTMT criterion.

Construct	AA	AB	AIA	LMD
AA				
AB	0.733			
AIA	0.485	0.537		
LMD	0.510	0.527	0.609	

**Table 3:** Discriminant validity evidence based on the Fornell–Larcker criterion.

Construct	AA	AB	AIA	LMD
AA	**0.844**			
AB	0.683	**0.849**		
AIA	0.448	0.505	**0.796**	
LMD	0.487	0.532	0.580	**0.748**

Bold diagonal values represent the square roots of the average variance extracted (AVE).

### 4.3 Structural model

Structural model collinearity was assessed using the inner VIF values for the predictors of each endogenous construct. As shown in Table 4, the values ranged from 1.000 to 1.703, indicating no substantial collinearity among the predictors in the specified model. These statistics concern structural model collinearity and were not used to establish the absence of common method bias (Hair et al., 2021; Sun et al., 2024).

**Table 4:** Collinearity test.

Construct	AA	AB	AIA	LMD
AA				
AB	1.518			
AIA	1.638	1.506		
LMD	1.703	1.506	1.000	

Table 5 and Figure 2 present the six estimated direct associations. All four hypothesized direct associations were statistically significant, supporting H1–H4. Large language model dependence was positively associated with artificial intelligence addiction (*β* = 0.580, 95% CI [0.477, 0.675], *p* < 0.001), supporting H1. Artificial intelligence addiction was positively associated with academic burnout (*β* = 0.296, 95% CI [0.148, 0.423], *p* < 0.001), supporting H2. Academic burnout was positively associated with academic anxiety (*β* = 0.569, 95% CI [0.432, 0.690], *p* < 0.001), supporting H3. Large language model dependence also showed a positive direct association with academic anxiety (*β* = 0.138, 95% CI [0.006, 0.278], *p* = 0.048), supporting H4, although the coefficient was small and the *p* value was close to the 0.05 threshold.

**Table 5:** Path hypothesis testing.

Hypothesis	Original sample (β)	2.50%	97.50%	*t*	*p*	*f* ^2^	Results
LMD → AIA	0.580	0.477	0.675	11.593	<0.001	0.506	Support
AIA → AB	0.296	0.148	0.423	4.206	<0.001	0.088	Support
AB → AA	0.569	0.432	0.690	8.564	<0.001	0.421	Support
LMD → AA	0.138	0.006	0.278	1.970	0.048	0.022	Support
LMD → AB	0.361	0.231	0.503	5.201	<0.001	0.131	Support
AIA → AA	0.081	−0.032	0.190	1.431	0.152	0.008	Not Support

**Figure 2:**
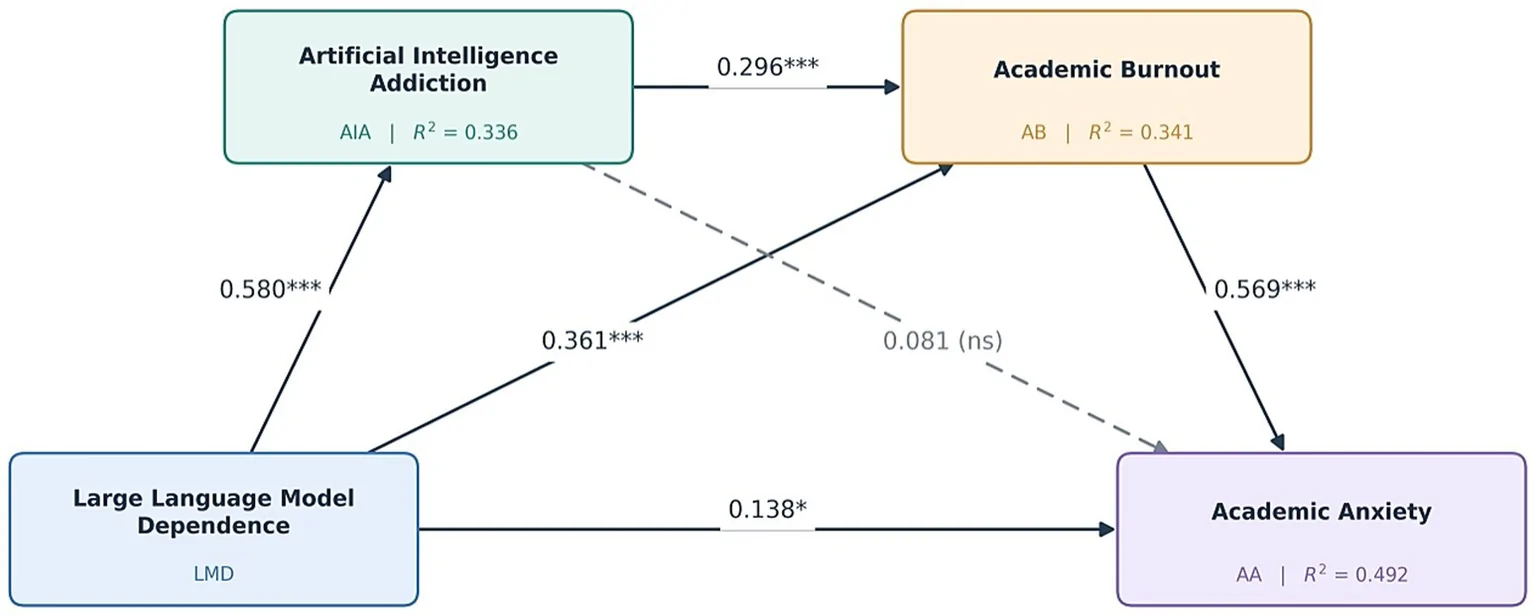
Structural model showing standardized path coefficients and explained variance. Values on arrows represent standardized path coefficients (β), and *R*^2^ values indicate the variance explained in each endogenous construct. Solid arrows indicate statistically significant associations; *p < 0.05; **p < 0.01; ***p < 0.001.

Two additional direct associations were estimated. Large language model dependence was positively associated with academic burnout (*β* = 0.361, 95% CI [0.231, 0.503], *p* < 0.001). In contrast, the direct association between artificial intelligence addiction and academic anxiety was not statistically significant (*β* = 0.081, 95% CI [−0.032, 0.190], *p* = 0.152). Neither covariate showed a statistically significant association with academic anxiety within the specified model (both *p* > 0.05). Both covariates were retained to account for potential demographic differences.

The *f*^2^ effect sizes reported in Table 5 indicated that large language model dependence made a large contribution to the explained variance in artificial intelligence addiction (*f*^2^ = 0.506). Academic burnout also made a large contribution to the explained variance in academic anxiety (*f*^2^ = 0.421). Artificial intelligence addiction and large language model dependence made small contributions to the explained variance in academic burnout (*f*^2^ = 0.088 and 0.131, respectively). For academic anxiety, the contribution of large language model dependence was small (*f*^2^ = 0.022), whereas that of artificial intelligence addiction was negligible (*f*^2^ = 0.008). Although the direct association between large language model dependence and academic anxiety reached statistical significance (*p* = 0.048), its small *f*^2^ indicates a limited incremental contribution to explained variance within the specified model.

Table 6 summarizes the explained variance of the endogenous constructs and the model-level fit indices. The model explained 49.2% of the variance in academic anxiety (*R*^2^ = 0.492), 34.1% in academic burnout (*R*^2^ = 0.341), and 33.6% in artificial intelligence addiction (*R*^2^ = 0.336). The SRMR value of 0.068 was below the reference value of 0.08, whereas the NFI value of 0.812 was below the reference value of 0.90. Thus, the fit indices provided mixed evidence regarding overall model fit. These findings warrant caution and do not support an unqualified conclusion that the model demonstrated acceptable overall fit.

**Table 6:** Explained variance, predictive assessment, and model fit.

Construct	*R* ^2^	*Q* ^2^	Model fit
AA	0.492	0.231	SRMR: 0.068
AB	0.341	0.276	NFI: 0.812
AIA	0.336	0.327	

The construct-level *Q*^2^_predict values were positive for academic anxiety (0.231), academic burnout (0.276), and artificial intelligence addiction (0.327), indicating lower prediction errors than those obtained using the training-sample mean benchmark. However, these values alone do not establish strong predictive performance; interpretation also requires the indicator-level prediction errors and their comparison with the linear model benchmark.

### 4.4 Statistical indirect associations

Table 7 presents the specific indirect associations estimated using bootstrapping with 5,000 resamples. The indirect association between large language model dependence and academic burnout through artificial intelligence addiction was statistically significant (*β* = 0.171, 95% CI [0.091, 0.243], *t* = 4.477, *p* < 0.001), supporting H5. The indirect association between large language model dependence and academic anxiety through academic burnout was also significant (*β* = 0.205, 95% CI [0.123, 0.303], *t* = 4.431, *p* < 0.001), supporting H6. Furthermore, the specific indirect association through artificial intelligence addiction followed by academic burnout was significant (*β* = 0.098, 95% CI [0.049, 0.147], *t* = 3.924, *p* < 0.001), supporting H7.

**Table 7:** Bootstrap estimates of specific indirect associations.

Relationship	Indirect effect	Direct effect	2.5%	97.5%	*t*	*p*
LMD → AIA → AB	0.171	0.361	0.091	0.243	4.477	<0.001
LMD → AB → AA	0.205	0.138	0.123	0.303	4.431	<0.001
LMD → AIA → AB → AA	0.098	0.138	0.049	0.147	3.924	<0.001
LMD → AIA → AA	0.047	0.138	−0.019	0.108	1.441	0.150
AIA → AB → AA	0.168	0.081	0.078	0.261	3.668	<0.001

Additional specific indirect associations were examined to provide a complete account of the estimated model. The indirect association between large language model dependence and academic anxiety through artificial intelligence addiction alone was not statistically significant (*β* = 0.047, 95% CI [−0.019, 0.108], *t* = 1.441, *p* = 0.150). In contrast, artificial intelligence addiction showed a significant indirect association with academic anxiety through academic burnout (*β* = 0.168, 95% CI [0.078, 0.261], *t* = 3.668, *p* < 0.001).

As reported in Table 5, the direct associations of large language model dependence with academic burnout and academic anxiety remained statistically significant within the specified model. These findings indicate the coexistence of direct and indirect associations, although not all specific indirect associations were supported.

Because the data were collected at a single time point, these findings represent statistical indirect associations within a theory-specified model rather than evidence of temporal ordering or causal mediation. The analysis cannot establish that artificial intelligence addiction precedes academic burnout or that academic burnout precedes academic anxiety. Reverse relationships and alternative explanations remain possible.

## 5 Discussion

This study examined direct and statistical indirect associations between large language model dependence and academic anxiety among nursing students, including those undertaking clinical placements. Large language model dependence was positively associated with artificial intelligence addiction, academic burnout, and academic anxiety, although its direct association with anxiety was small. Significant specific indirect associations with academic anxiety were identified through academic burnout and through artificial intelligence addiction followed by academic burnout. The indirect association through artificial intelligence addiction alone was not statistically significant. These findings support some of the hypothesized associations while highlighting the need to distinguish the specific indirect relationships within the model.

The positive association between large language model dependence and artificial intelligence addiction is consistent with prior research on problematic technology use, which suggests that repeated reliance on digital technologies may be associated with maladaptive patterns of use when individuals experience reduced self-regulation and difficulty controlling their engagement (Canbeldek Erol et al., 2026; Li and Jiang, 2025). This finding is also consistent with the recent research on the dependence of large language models, which conceptualizes dependence as a shift from functional assistance to dependence when users increasingly expect AI systems to meet their academic, cognitive or work-related needs (Li et al., 2025). In this study, nursing students who are more dependent on large language models are also more likely to report a higher degree of AI addiction. This supports the view that frequent or instrumental AI use is not necessarily problematic, but excessive reliance may be associated with greater vulnerability to poorly regulated AI use. Compared with earlier studies that mainly emphasized the usefulness, acceptability or effectiveness of AI tools, the findings of this study add a more cautious perspective, indicating that AI-related dependence may also be associated with maladaptive patterns of AI use.

The observed association between AI addiction and academic burnout is also generally consistent with the studies of problematic Internet use, technology overuse and academic burnout. Previous studies have shown that overuse of digital tools has been associated with disrupted learning routines, reduce active academic participation, and greater psychological pressure (Farghaly Abdelaliem et al., 2022; Ibrahim et al., 2025; Sağlam and Kalanlar, 2025). Similarly, this study found that AI addiction was positively correlated with academic burnout of nursing-related participants. This result shows that maladaptive AI use may not only reflect the high frequency of use, but also be related to the reduction of learning motivation and emotional exhaustion. However, this study is different from many previous studies, which focuses on AI addiction rather than general digital addiction or Internet addiction (El-Gazar et al., 2024; Gu et al., 2023; Wei et al., 2025; Zhou et al., 2022). This difference is significant because AI tools, especially large language models, are interactive, responsive and task oriented. Therefore, users may not only rely on entertainment or social interaction, but also rely on AI assisted thinking, writing and problem solving. This may result in different forms of academic vulnerability, especially in areas such as nursing that require independent judgment and professional reasoning.

The significant correlation between academic burnout and academic anxiety is consistent with previous studies, indicating that academic burnout is closely related to the negative psychological outcomes of students and healthcare-related populations. Burnout usually manifests as emotional exhaustion, reduced participation and reduced academic efficacy, all of which may be linked to greater concerns about academic achievement and perceived inadequacy (Alzahrani and Albloushi, 2025). In this regard, the results of this study support the framework of stress-related psychological resources, which indicates that when anxiety is more likely to occur, individuals believe that academic demands exceed their available coping resources. For nursing students, academic and clinical learning environments may involve sustained stress, performance expectations, and emotional needs (Sarfika et al., 2025). Therefore, when the level of academic burnout increases, learning tasks may be considered more threatening or more difficult to manage, which may be related to stronger academic anxiety. However, unlike longitudinal studies, which can examine changes in time, the current cross-sectional design only supports the association between burnout and anxiety, rather than confirming that burnout leads to anxiety over time.

The direct association between large language model dependence and academic anxiety provides a modest extension to the existing literature. Previous studies often discussed AI tools as resources to improve learning efficiency, reduce workload and support academic achievement (Harrington et al., 2025; Wang et al., 2025). This study does not deny these potential benefits, but it shows that excessive dependence on large language models may coexist with higher academic anxiety (Jukiewicz, 2025). One possible explanation is that individuals who rely heavily on reactions generated by AI may lack confidence in their academic abilities, especially when facing tasks requiring independent reasoning, critical assessment or professional judgment (Tian and Zhang, 2025). This finding is particularly relevant to nursing education and practice. In nursing education and practice, ability depends not only on obtaining information, but also on interpreting, applying and evaluating knowledge in complex situations. Compared with the early studies focusing on the positive functions of AI assisted learning (Guizani et al., 2025), the direct association between large language model dependence and academic anxiety was statistically significant but small in magnitude. Therefore, this result should not be overinterpreted as indicating that large language model dependence is a strong independent predictor of academic anxiety. Instead, the findings suggest that large language model dependence may be one modest correlate of anxiety, and its relevance may be better understood in combination with problematic AI use and academic burnout. However, this result should be carefully explained: this finding does not indicate that extensive use of language models leads to anxiety; rather, it suggests that dependence on such tools may be meaningfully associated with academic anxiety in nursing-related populations.

The significant statistical indirect associations are compatible with the proposed theoretical ordering, but alternative explanations remain possible. Students experiencing greater academic anxiety may seek more AI assistance to manage uncertainty or concerns about performance. Similarly, students experiencing academic burnout may find independent task completion more demanding and rely more heavily on AI support (Zhang et al., 2024). Academic pressure and pre-existing psychological distress may also be associated with all four constructs, contributing to their observed relationships. These reverse relationships and shared influences were not tested in the present study. The findings therefore cannot distinguish the proposed ordering from alternative models, which should be examined using longitudinal data and explicit comparisons of competing explanations.

### 5.1 Theoretical implications

This study offers several theoretical implications for the application of artificial intelligence, academic psychology and nursing education. First, the literature on the use of large language models is expanded by shifting attention from the functional value of AI tools to their possible psychological and behavioral factors. Previous studies mainly focused on the usefulness, acceptability, effectiveness and learning support function of AI in education and professional environment. On the contrary, this study suggests that large language model dependence should also be regarded as a psychological structure, which may be related to maladaptive AI use, burnout and academic anxiety. This provides a more balanced theoretical understanding of AI interaction, indicating that when AI tools are overused or mismanaged, their educational benefits may coexist with potential psychological vulnerability (Garzón et al., 2025; Labrague et al., 2025).

Second, this study contributes to the literature by clarifying the distinction between large language model dependence and artificial intelligence addiction. Although both constructs belong to the broader domain of problematic AI-related use, they capture different psychological and behavioral meanings. LMD reflects reliance on a specific generative AI tool for academic and work-related task completion, whereas AI addiction reflects a dysregulated pattern of AI use involving excessive engagement, reduced control, and continued use despite possible negative consequences (Alshowkan and Shdaifat, 2025; Ayed et al., 2025).

Third, the study integrates perspectives on problematic technology use and social stress to examine (Aneshensel, 1992) statistical indirect associations between large language model dependence and academic anxiety. Artificial intelligence addiction represents difficulties regulating AI engagement, whereas academic burnout represents study-related exhaustion (Hwang and Kim, 2022). The findings were consistent with the specified indirect association involving these two constructs. However, they do not demonstrate a behavioral-to-psychological progression or establish that either construct causally mediates the association. The model provides a basis for developing and testing competing explanations in future longitudinal research. Research in other populations has examined coping self-efficacy and emotion regulation as intermediate constructs in associations between mindfulness exercise and mental health (Yan et al., 2026c). Although these findings concern older adults and different exposures, they suggest additional psychological resources that could be examined in future models of AI-related engagement and academic distress.

Finally, the study extends the examination of AI-related dependence and academic distress to nursing students, including those undertaking clinical placements. The specified model identifies statistical indirect associations involving artificial intelligence addiction and academic burnout in this educational context. Its theoretical contribution lies in articulating relationships that can be examined further, rather than establishing a temporal sequence or causal mechanism. Future research should compare alternative orderings and investigate whether these associations vary across stages of nursing education and clinical placement experience.

### 5.2 Practical implications

The findings identify issues that merit attention in nursing education, although the study did not evaluate the effectiveness of educational or psychological interventions. Nursing programs may consider incorporating AI literacy into classroom teaching and clinical placement preparation, including critical evaluation of AI-generated information, verification against authoritative sources, privacy protection, and appropriate disclosure of AI assistance (Hardie et al., 2026; Simms, 2025). Assignments requiring students to explain their own reasoning and evaluate AI outputs could be explored as ways to support reflective engagement. Their effects on independent learning and problematic AI reliance require direct evaluation.

The small direct association between large language model dependence and academic anxiety suggests that dependence should be considered alongside academic burnout and difficulties regulating AI engagement. Educators may consider discussing students’ reasons for relying on AI (Sağlam and Kalanlar, 2025), difficulties completing tasks independently, and experiences of academic exhaustion. Such discussions could inform decisions about academic guidance or referral to existing support services. The measures used in this study do not establish clinical diagnoses or thresholds for intervention.

Academic workload, clinical placement demands, and access to mentoring and psychological support also warrant consideration (Gillett-Swan and Grant-Smith, 2020; Yan et al., 2026a, 2026b). Potential approaches include reviewing workload expectations, strengthening supervisory support, and improving access to counseling and peer support. These recommendations represent directions for practice and future evaluation; the present findings do not demonstrate that changing AI use or reducing burnout will improve academic anxiety.

### 5.3 Limitations and future research directions

This study has several limitations, which should be taken into account when interpreting the findings. First, the cross-sectional design limits the ability to draw causal or temporal conclusions. Although the proposed model is based on theoretical reasoning, the data were collected at a single point in time. Therefore, the relationship among large language model dependence, AI addiction, academic burnout and academic anxiety should be interpreted as associations rather than causality. In particular, mediation results should be understood as statistical indirect associations rather than evidence of temporal or causal mechanisms. Future research should use longitudinal, cross-lagged, diary based or experimental designs to examine whether large language model dependencies precede changes in problematic AI use, burnout and anxiety over time.

Second, the self-report questionnaire from the same participants is used to measure all variables, which may introduce recall bias, social desirability bias, response bias and common method variance methods. Although this study used procedural controls and statistical diagnosis to reduce and examine common method bias, these methods can not completely exclude the impact of data collection from the same source. Future research should adopt multi-source and multi method design, such as combining self-report measurement with AI use behavior indicators, learning platform records, academic performance data, peer or mentor evaluation, and qualitative interviews. These methods will provide a more comprehensive understanding of how AI is related to dependence and academic and psychological adjustment.

The sample comprised nursing students, including those undertaking clinical placements. Students at different stages of nursing education may differ in academic demands, clinical exposure, and patterns of AI use. The present analysis did not establish whether the observed associations varied across these educational stages. Future studies should record educational and placement status in greater detail and examine potential differences. The findings should not be generalized directly to practicing nurses or other professional populations.

Fourth, although this study distinguishes large language model dependence and AI addiction at the conceptual and empirical levels, the two structures are still closely related in the broader field of AI related use problems. Large language model dependence reflects the dependence on academic, cognitive or work-related task help generated by the large language model, while AI addiction reflects a more dysfunctional pattern of AI use, including excessive engagement, impaired control and continuous use, although it may have negative consequences. Future research should further refine the conceptual boundaries between these structures, and examine whether they represent distinct dimensions, sequential stages or overlapping components of maladaptive AI use.

Fifth, convenience sampling through institutional and online communication channels may have introduced selection bias. Students who could access the recruitment channels, were willing to complete an online survey, or had greater interest in AI may have been more likely to participate. Their AI-related experiences and psychological characteristics may differ from those of nonparticipants. Furthermore, the sample was restricted to nursing students in Guangdong Province, limiting generalizability to other disciplines, regions, and cultural settings. Future studies should use more diverse recruitment strategies and examine these associations in independent samples.

Finally, demographic adjustment was limited to including age category and sex as predictors of academic anxiety. Although neither covariate was statistically significant, residual confounding cannot be excluded. These covariates were not included in the equations predicting artificial intelligence addiction or academic burnout. Age was recorded in grouped categories and entered as a single ordinal predictor, which assumes a linear association across categories and does not capture within-group age differences. AI-use frequency, duration, and purpose were not measured, so their potential confounding influence could not be evaluated. Future studies should collect more detailed demographic and AI-use information and examine the sensitivity of the estimated associations to alternative covariate specifications.

## 6 Conclusion

Large language model dependence was positively associated with academic anxiety among nursing students, including those undertaking clinical placements. The direct association was small, while significant specific indirect associations were identified through academic burnout and through artificial intelligence addiction followed by academic burnout. The indirect association through artificial intelligence addiction alone was not statistically significant, highlighting the importance of distinguishing the specific associations within the model.

These findings contribute to understanding the relationships among AI-related dependence, problematic AI use, and academic distress in nursing education. However, the cross-sectional design does not establish temporal ordering or causal mediation, and the mixed model fit evidence warrants cautious interpretation. Responsible AI use, independent learning, and academic well-being merit attention in nursing education, while longitudinal and intervention studies are needed to clarify these relationships and evaluate appropriate support strategies.

## Data Availability

The original contributions presented in the study are included in the article/supplementary material, further inquiries can be directed to the corresponding author.

## References

[ref1] Ali L. A. E. H. Ali M. I. E. B. Ali S. M. A. Nashwan A. J. (2025). Smartphone dependency, digital amnesia, and somatic symptoms among nursing students: the challenge of artificial intelligence. BMC Nurs. 24:599. doi: 10.1186/s12912-025-03228-0, 40420144 PMC12107735

[ref2] Al-Obaydi L. H. Pikhart M. (2026). Artificial intelligence addiction: exploring the emerging phenomenon of addiction in the AI age. AI Soc. 41, 1577–1593. doi: 10.1007/s00146-025-02535-z

[ref3] Alshowkan A. Shdaifat E. (2025). Impact of AI literacy on well-being among nursing students—mediating roles of empowerment and anxiety: cross-sectional study. JMIR Nurs. 8:e79789. doi: 10.2196/79789, 41428941 PMC12721487

[ref4] Alzahrani E. Albloushi M. (2025). Undergraduate nursing students' academic burnout: a concept analysis to inform future research and interventions. J. Prof. Nurs. 63, 58–63. doi: 10.1016/j.profnurs.2025.12.004, 41796562

[ref5] Andargeery S. Y. Altaweel E. A. Alkorbi F. S. Alyousef L. A. Alanazi B. F. Idriss S. H. . (2025). Nursing students’ academic burnout: its prevalence and association with self-efficacy, psychological distress, and quality of learning experience. BMC Nurs. 24:631. doi: 10.1186/s12912-025-03242-2, 40462100 PMC12131394

[ref6] Aneshensel C. S. (1992). Social stress: theory and research. Annu. Rev. Sociol. 18, 15–38.

[ref7] Ayed A. Ejheisheh M. A. Al-Amer R. Aqtam I. Ali A. M. Othman E. H. . (2025). Insights into the relationship between anxiety and attitudes toward artificial intelligence among nursing students. BMC Nurs. 24:812. doi: 10.1186/s12912-025-03490-2, 40597186 PMC12211391

[ref8] Bastani H. Bastani O. Sungu A. Ge H. Kabakcı Ö. Mariman R. (2025). Generative AI without guardrails can harm learning: evidence from high school mathematics. Proc. Natl. Acad. Sci. 122:e2422633122. doi: 10.1073/pnas.2422633122, 40560616 PMC12232635

[ref9] Bülbül K. Odacı H. (2025). Akademik kaygı ölçeği: Bir ölçek geliştirme çalışması. Dokuz Eylül Üniversitesi Buca Eğitim Fakültesi Dergisi 63, 477–499. doi: 10.53444/deubefd.1531518

[ref10] Canbeldek Erol M. Erol A. Erol M. Budak Topaloğlu K. S. (2026). Determinants of artificial intelligence addiction: anxiety, self-efficacy, attitudes, literacy, perceived ease of use, behavioral intention, and perceived usefulness. Int. J. Hum.-Comput. Interact., 1–24. doi: 10.1080/10447318.2026.2619035

[ref11] Chang B. Hou J. (2022). The association between perceived risk of COVID-19, psychological distress, and internet addiction in college students: an application of stress process model. Front. Psychol. 13:898203. doi: 10.3389/fpsyg.2022.898203, 35795419 PMC9252417

[ref12] Cohen J. (2013). Statistical Power Analysis for the Behavioral Sciences. routledge.

[ref13] El-Gazar H. E. Elgohari H. Loutfy A. Shawer M. El-Monshed A. H. Abou Zeid M. A. G. . (2024). Does internet addiction affect the level of emotional intelligence among nursing students? A cross-sectional study. BMC Nurs. 23:555. doi: 10.1186/s12912-024-02191-6, 39135017 PMC11321225

[ref14] Elsamen A. A. Fotiadis A. Alalwan A. A. Huan T.-C. (2025). Enhancing pro-environmental behavior in tourism: integrating attitudinal factors and norm activation theory. Tour. Manag. 109:105155. doi: 10.1016/j.tourman.2025.105155

[ref15] El-Sayed A. A. I. Alsenany S. A. Asal M. G. R. Alasqah I. (2025). Development and validation of artificial intelligence addiction scale for researchers: a methodological study. J. Nurs. Manag. 2025:8458533. doi: 10.1155/jonm/8458533, 41424990 PMC12714078

[ref16] Elshaer I. A. Azazz A. M. (2025). The impacts of large language model addiction on university students’ mental health: gender as a moderator. Algorithms 18:789. doi: 10.3390/a18120789

[ref17] Fard F. S. Asayesh H. Hosseini M. H. M. Sepahvandi M. (2020). Motivation, self-efficacy, stress, and academic performance correlation with academic burnout among nursing students. J. Nurs. Midwifery Sci. 7:e140800, 88–93.

[ref18] Farghaly Abdelaliem S. M. Dator W. L. T. Sankarapandian C. (2022). The relationship between nursing students’ smart devices addiction and their perception of artificial intelligence. Healthcare. 11:110. 10.3390/healthcare11010110 PMC9819298 36611570

[ref19] García-Velasco L. Alcoceba-Herrero I. García S. López M. Albertos-Muñoz I. Castro M.-J. . (2025). Assessing anxiety and stress levels in undergraduate nursing students during their clinical placements: a quasi-experimental study. BMC Nurs. 24:620. doi: 10.1186/s12912-025-03264-w, 40450309 PMC12125880

[ref20] Garzón J. Patiño E. Marulanda C. (2025). Systematic review of artificial intelligence in education: trends, benefits, and challenges. Multimodal Technol. Interact. 9:84. doi: 10.3390/mti9080084

[ref21] Ghattas A. H. S. El-Ashry A. M. (2024). Perceived academic anxiety and procrastination among emergency nursing students: the mediating role of cognitive emotion regulation. BMC Nurs. 23:670. doi: 10.1186/s12912-024-02302-3, 39300404 PMC11414069

[ref22] Gillett-Swan J. Grant-Smith D. (2020). Addressing mentor wellbeing in practicum placement mentoring relationships in initial teacher education. Int. J. Mentoring Coaching Educ. 9, 393–409. doi: 10.1108/ijmce-02-2020-0007

[ref23] Gu J. Wu P. Luo Y. He X. Fu L. Liu H. . (2023). Internet addiction, loneliness, and academic burnout among Chinese college students: a mediation model. Front. Psych. 14:1176596. doi: 10.3389/fpsyt.2023.1176596, 37663601 PMC10469855

[ref24] Guizani S. Mazhar T. Shahzad T. Ahmad W. Bibi A. Hamam H. (2025). A systematic literature review to implement large language model in higher education: issues and solutions. Discov. Educ. 4:35. doi: 10.1007/s44217-025-00424-7

[ref25] Gunawan J. Aungsuroch Y. Montayre J. (2024). ChatGPT integration within nursing education and its implications for nursing students: a systematic review and text network analysis. Nurse Educ. Today 141:106323. doi: 10.1016/j.nedt.2024.106323, 39068726

[ref26] Hair J. F. Hult G. T. M. Ringle C. M. Sarstedt M. Danks N. P. Ray S. (2021). Partial Least Squares Structural Equation Modeling (PLS-SEM) Using R: A Workbook. Cham, Switzerland: Springer Nature.

[ref27] Hair J. F. Risher J. J. Sarstedt M. Ringle C. M. (2019). When to use and how to report the results of PLS-SEM. Eur. Bus. Rev. 31, 2–24. doi: 10.1108/ebr-11-2018-0203

[ref28] Hardie P. Darley A. Derwin R. Eustace-Cook J. Kearns S. Mc Brien B. . (2026). Applications, attitudes and ethical considerations of generative artificial intelligence (gen AI) in nursing education: a scoping review. BMC Nurs. 25:148. doi: 10.1186/s12912-025-04253-9, 41545825 PMC12892504

[ref29] Harrington J. Booth R. G. Jackson K. T. (2025). Large language models in nursing education: concept analysis. JMIR Nurs. 8:e77948. doi: 10.2196/77948, 40845300 PMC12373302

[ref30] Hobensack M. von Gerich H. Vyas P. Withall J. Peltonen L.-M. Block L. J. . (2024). A rapid review on current and potential uses of large language models in nursing. Int. J. Nurs. Stud. 154:104753. doi: 10.1016/j.ijnurstu.2024.104753 38560958

[ref31] Hwang E. Kim J. (2022). Factors affecting academic burnout of nursing students according to clinical practice experience. BMC Med. Educ. 22:346. doi: 10.1186/s12909-022-03422-7, 35524256 PMC9077997

[ref32] Ibrahim R. K. Khaled M. Almansoori M. Almazrouei M. Ashraf A. Alahmedi S. H. . (2025). Screen time and stress: understanding how digital burnout influences health among nursing students. BMC Nurs. 24:990. doi: 10.1186/s12912-025-03621-9, 40730989 PMC12309007

[ref33] Jan S. Parveen A. (2024). Construction and validation of academic burnout scale. Discov. Psychol. 4:104. doi: 10.1007/s44202-024-00201-x

[ref34] Jukiewicz M. (2025). How generative artificial intelligence transforms teaching and influences student wellbeing in future education. Front. Educ. 10:1594572. doi: 10.3389/feduc.2025.1594572

[ref35] Kong L.-N. Yao Y. Chen S.-Z. Zhu J.-L. (2023). Prevalence and associated factors of burnout among nursing students: a systematic review and meta-analysis. Nurse Educ. Today 121:105706. doi: 10.1016/j.nedt.2022.105706, 36577286

[ref36] Labrague L. J. Sabei S. A. Yahyaei A. A. (2025). Artificial intelligence in nursing education: a review of AI-based teaching pedagogies. Teach. Learn. Nurs. 20, 210–221. doi: 10.1016/j.teln.2025.01.019

[ref37] Li Y. Jiang J. (2025). How time pressure intensifies artificial intelligence addiction among graduate students: exploring the role of academic control deprivation and self-reflexivity across engagement profiles. High. Educ., 1–19.

[ref38] Li Z. Zhang Z. Wang M. Wu Q. (2025). From assistance to reliance: development and validation of the large language model dependence scale. Int. J. Inf. Manag. 83:102888. doi: 10.1016/j.ijinfomgt.2025.102888

[ref39] Liu X. Liu Y. Dai Y. Fu J. (2026). Academic stress and university students’ dependency on generative artificial intelligence: a multiple mediation model using PLS-SEM. BMC Psychol. 14. doi: 10.1186/s40359-026-03986-9, 41547828 PMC12895799

[ref40] Ma Y. Liu T. Qi J. Gan Y. Cheng Q. Xiao M. . (2025). Facilitators and barriers of large language model adoption among nursing students: a qualitative descriptive study. J. Adv. Nurs. 81, 4856–4870. doi: 10.1111/jan.16655, 39755439

[ref41] Maral S. Naycı N. Bilmez H. Erdemir E. İ. Satici S. A. (2025). Problematic ChatGPT use scale: AI-human collaboration or unraveling the dark side of ChatGPT. Int. J. Ment. Health Addict., 1–27.

[ref42] Mohamed N. A. Ali S. O. Ehrahim E. E. E. Ahmed A. L. Wahba A. M. (2024). Predictors of academic and clinical stress among nursing students. SAGE Open Nurs. 10:23779608241290392. doi: 10.1177/23779608241290392, 39469726 PMC11514110

[ref43] Montag C. Yang H. Wu A. M. Ali R. Elhai J. D. (2025). The role of artificial intelligence in general, and large language models specifically, for understanding addictive behaviors. Ann. N. Y. Acad. Sci. 1548, 5–11. doi: 10.1111/nyas.15337, 40302174 PMC12220279

[ref44] Olson N. Oberhoffer-Fritz R. Reiner B. Schulz T. (2025). Stress, student burnout and study engagement–a cross-sectional comparison of university students of different academic subjects. BMC Psychol. 13:293. doi: 10.1186/s40359-025-02602-6, 40128867 PMC11931745

[ref45] Podsakoff P. M. MacKenzie S. B. Lee J.-Y. Podsakoff N. P. (2003). Common method biases in behavioral research: a critical review of the literature and recommended remedies. J. Appl. Psychol. 88, 879–903. doi: 10.1037/0021-9010.88.5.879, 14516251

[ref46] Qin Z. Yang G. Lin Z. Ning Y. Chen X. Zhang H. . (2025). The impact of academic burnout on academic achievement: a moderated chain mediation effect from the stimulus-organism-response perspective. Front. Psychol. 16:1559330. doi: 10.3389/fpsyg.2025.1559330, 40538475 PMC12178059

[ref47] Sağlam R. K. Kalanlar B. (2025). Living with and without AI: a mixed-methods study on AI usage, addiction, and 'AIlessphobia' in nursing students. Nurse Educ. Pract. 88:104530. doi: 10.1016/j.nepr.2025.104530, 40896955

[ref48] Sallam M. (2023). ChatGPT utility in healthcare education, research, and practice: systematic review on the promising perspectives and valid concerns. Healthcare. 11:887. 10.3390/healthcare11060887 PMC10048148 36981544

[ref49] Sarfika R. Azzahra W. Ananda Y. Saifudin I. M. M. Y. Abdullah K. L. (2025). Academic burnout among nursing students: the role of stress, depression, and anxiety within the demand control model. Teach. Learn. Nurs. 20, 253–260. doi: 10.1016/j.teln.2025.02.019

[ref50] Sarstedt M. Ringle C. M. Hair J. F. (2021). “Partial least squares structural equation modeling,” in Handbook of Market Research, (Cham, Switzerland: Springer), 587–632.

[ref51] Seshabela H. D. Shakwane S. (2024). Impact of anxiety-related conditions on nursing students’ academic excellence. Curationis 47, e1–e10. doi: 10.4102/curationis.v47i1.2571, 39494647 PMC11538042

[ref52] Sharifi Kelarijani A. Safdari A. Golitaleb M. (2024). Every coin has two sides: ChatGPT poses a potential threat to nursing students' education. Front. Med. 11:1415067. doi: 10.3389/fmed.2024.1415067, 39114822 PMC11303222

[ref53] Simms R. C. (2025). Generative artificial intelligence (AI) literacy in nursing education: a crucial call to action. Nurse Educ. Today 146:106544. doi: 10.1016/j.nedt.2024.106544, 39708405

[ref54] Sun M. Piao M. Jia Z. (2024). The impact of alexithymia, anxiety, social pressure, and academic burnout on depression in Chinese university students: an analysis based on SEM. BMC Psychol. 12:757. doi: 10.1186/s40359-024-02262-y, 39695783 PMC11657414

[ref55] Tian J. Zhang R. (2025). Learners' AI dependence and critical thinking: the psychological mechanism of fatigue and the social buffering role of AI literacy. Acta Psychol. 260:105725. doi: 10.1016/j.actpsy.2025.105725, 41076923

[ref56] Wang Y. Zhang T. Yao L. Seedhouse P. (2025). A scoping review of empirical studies on generative artificial intelligence in language education. Innov. Lang. Learn. Teach., 1–28. doi: 10.1080/17501229.2025.2509759, 37339054

[ref57] Wei Z. Hassan N. C. Hassan S. A. Ismail N. Gu X. Dong J. (2025). The relationship between internet addiction and academic burnout in undergraduates: a chain mediation model. BMC Public Health 25:1523. doi: 10.1186/s12889-025-22719-y, 40275249 PMC12020274

[ref58] Yan Y. Ye Q. Dong J. Ye S. Li T. (2026c). Mindfulness exercise and mental health in older adults: a cross-sectional study of the mediating roles of coping self-efficacy and emotion regulation. Clin. Interv. Aging 21, 1–18. doi: 10.2147/CIA.S622746, 42631288 PMC13496253

[ref59] Yan Y. Ye Q. Li T. Yuan Y. Dong J. (2026a). Depression and mental health in late adolescents: the sequential mediating roles of quality of life and sleep regularity. BMC Psychiatry 26:316. doi: 10.1186/s12888-026-07944-y, 41803743 PMC13085598

[ref60] Yan Y. Ye Q. Li T. Yuan Y. Dong J. (2026b). Mindfulness exercise and quality of life among nursing students: the mediating roles of self-control and internet addiction. BMC Nurs. 25:501. doi: 10.1186/s12912-026-04669-x, 42001094 PMC13227776

[ref61] Yankouskaya A. Liebherr M. Ali R. (2025). Can ChatGPT be addictive? A call to examine the shift from support to dependence in AI conversational large language models. Hum. Centric Intell. Syst. 5, 77–89. doi: 10.1007/s44230-025-00090-w

[ref62] Ye X. Shan X. Tu Y. Zhang Y. (2025). Examining the efficacy of large language models for mitigating depression and anxiety among Chinese students: a randomized controlled trial. CIN 10:1097. 10.1097/CIN.0000000000001349 40704906

[ref63] Zhang S. Zhao X. Zhou T. Kim J. H. (2024). Do you have AI dependency? The roles of academic self-efficacy, academic stress, and performance expectations on problematic AI usage behavior. Int. J. Educ. Technol. High. Educ. 21:34. doi: 10.1186/s41239-024-00467-0

[ref64] Zhou Z. Liu H. Zhang D. Wei H. Zhang M. Huang A. (2022). Mediating effects of academic self-efficacy and smartphone addiction on the relationship between professional attitude and academic burnout in nursing students: a cross-sectional study. Nurse Educ. Today 116:105471. doi: 10.1016/j.nedt.2022.105471, 35834868

[ref65] Zhu P. Xu T. Xu H. Ji Q. Wang W. Qian M. . (2023). Relationship between anxiety, depression and learning burnout of nursing undergraduates after the COVID-19 epidemic: the mediating role of academic self-efficacy. Int. J. Environ. Res. Public Health 20:4194. doi: 10.3390/ijerph20054194, 36901200 PMC10002455

